# CRISPR/Cas9-Mediated Knockout of *OsHSBP1* Confers Heat Tolerance to Bacthom 7 Elite Rice Cultivar

**DOI:** 10.3390/biotech15010013

**Published:** 2026-02-04

**Authors:** Phuong Duy Nguyen, Van Thi Pham, Ha Thanh Nguyen, Khoa Dang Dang, Tu Tuan Tran, Dai Lan Tran, Thanh Duc Nguyen, Thao Duc Le, Xuan Hoi Pham, Xuan Dang Tran, Quyen Le Cao

**Affiliations:** 1 Department of Molecular Pathology, Institute of Agricultural Genetics, Vietnam Academy of Agricultural Sciences, Hanoi 100000, Vietnam; phuongnd.bio@gmail.com (P.D.N.); maiphuongvan0209@gmail.com (V.T.P.); hathanh73nguyen@gmail.com (H.T.N.); daitl@hnue.edu.vn (D.L.T.); nguyenthanhduc0212@yahoo.com.vn (T.D.N.); leducthao246@gmail.com (T.D.L.); xuanhoi.pham@gmail.com (X.H.P.); 2 Center for Scientific Research and Application, Lac Hong University, Dong Nai 430000, Vietnam; khoadd@lhu.edu.vn; 3 Admission Office of Vietnam Academy of Science and Technology, Vietnam Academy of Science and Technology, Hanoi 100000, Vietnam; tuantutran166@gmail.com; 4 Faculty of Biology, Hanoi National University of Education, Hanoi 100000, Vietnam; 5 Graduate School of Innovation and Practice for Smart Society, Hiroshima University, Higashi-Hiroshima 739-8529, Japan; 6 Graduate School of Advanced Science and Engineering, Hiroshima University, 1-5-1 Kagamiyama, Higashi-Hiroshima 739-8529, Japan; 7 Center for the Planetary Health and Innovation Science (PHIS), The IDEC Institute, Hiroshima University, Higashi-Hiroshima 739-8529, Japan; 8 Graduate School of Integrated Sciences for Life, Hiroshima University, Higashi-Hiroshima 739-8529, Japan

**Keywords:** Bacthom 7, CRISPR/Cas9, heat tolerance, HSP, *OsHSBP1*

## Abstract

This study investigates the functional role of *OsHSBP1*, a heat shock factor-binding protein, in regulating abiotic stress tolerance in rice, with the aim of enhancing climate resilience in the elite indica cultivar Bacthom 7 (BT7). Using Clustered Regularly Interspaced Short Palindromic Repeats/CRISPR-associated protein 9 (CRISPR/Cas9) genome editing, we generated transgene-free homozygous knockout lines targeting *OsHSBP1* and evaluated their physiological, biochemical, and agronomic responses under heat stress. Mutant lines exhibited markedly improved tolerance to both stresses, with survival rates reaching 43–46% under heat stress, compared to near-zero in wildtype plants. Enhanced tolerance was associated with significantly increased catalase and peroxidase activities and reduced oxidative damage, including lower malondialdehyde content and decreased superoxide accumulation. Despite these stress-related advantages, the knockout lines showed minimal differences in key agronomic traits under normal growing conditions, with comparable plant height, tillering ability, grain yield, and amylose content relative to the wildtype. These results demonstrate that *OsHSBP1* functions as a negative regulator of abiotic stress tolerance in rice, and its knockout enhances resilience without compromising yield potential. The study highlights *OsHSBP1* as a promising target for precision breeding of climate-resilient rice cultivars.

## 1. Introduction

Climate change has emerged as one of the most pressing challenges for global food security, with rising temperatures posing significant threats to agricultural productivity [1]. Rice (*Oryza sativa* L.), as a staple food crop feeding more than half of the world’s population, is particularly vulnerable to these environmental stresses [2]. Heat stress (HS) occurring during critical developmental stages can severely reduce rice grain yield by 10–50%, making the development of stress-tolerant varieties an urgent priority for sustainable agriculture [3].

To cope with heat stress, plants have evolved sophisticated molecular mechanisms centered on the heat shock response (HSR). Central to this response is the dynamic regulation of heat shock factors (HSFs) and heat shock proteins (HSPs), which maintain protein homeostasis and protect against heat-induced cellular damage [4,5]. The HSR is tightly controlled by negative regulators, including heat shock factor binding proteins (HSBPs). HSBPs attenuate HSF activity by binding to active HSF trimers, preventing excessive or prolonged transcriptional activation [6,7,8]. In rice, two HSBP genes—*OsHSBP1* and *OsHSBP2*—have been identified, with *OsHSBP1* displaying constitutive expression and upregulation during recovery after HS [9,10]. Previous RNA interference studies suggested that partial suppression of *OsHSBP1* enhances HSP expression and heat tolerance [10]; however, these RNAi lines exhibited developmental defects including reduced seed setting rate, raising concerns about off-target effects or the incomplete nature of RNAi-mediated gene suppression. These findings underscore the potential of targeting *OsHSBP1* to enhance heat tolerance, but also highlight the need for more precise genetic approaches to achieve complete gene knockout while avoiding pleiotropic effects on development.

Genome editing technologies, particularly CRISPR/Cas9, have enabled precise modification of endogenous genes without introducing foreign DNA [11]. Unlike conventional transgenic approaches, CRISPR/Cas9-mediated knockouts create targeted mutations indistinguishable from natural variations. This offers significant advantages for regulatory approval and public acceptance [12]. Several successful applications of CRISPR/Cas9 in rice have demonstrated its potential for enhancing abiotic stress tolerance. For instance, knockout of *OsERA1* conferred drought tolerance without compromising plant growth under normal conditions [13], while mutations in *OsCNGC14* and *OsCNGC* genes improved HS tolerance [14,15]. These studies highlight the utility of precision gene editing for dissecting the molecular basis of stress responses and developing climate-resilient rice varieties.

Despite these advances, critical questions remain unresolved regarding *OsHSBP1* function in rice heat stress tolerance. Previous RNAi studies achieved only partial gene suppression [10], leaving uncertainty about the consequences of complete loss-of-function. Moreover, while RNAi-mediated knockdown enhanced heat tolerance at the seedling stage, it also impaired seed development [10], raising concerns about potential pleiotropic effects and yield trade-offs. Whether complete and stable knockout of *OsHSBP1* through CRISPR/Cas9 can enhance seedling heat tolerance without negatively affecting normal plant growth and agronomic traits remains unknown. A comprehensive evaluation combining seedling stress responses with agronomic performance under non-stress conditions is needed to determine the breeding value of *OsHSBP1* knockout alleles.

Addressing these questions requires investigation in elite commercial cultivars that are widely grown yet remain vulnerable to heat stress. BT7 is a high-yielding indica rice cultivar widely cultivated in northern Vietnam, known for its excellent grain quality [16]. However, BT7 remains susceptible to high temperature condition that are becoming increasingly common in northern Vietnam due to climate change. Improving the stress resilience of BT7 while maintaining its desirable agronomic characteristics would provide significant benefits to local farmers and contribute to food security in Vietnam.

In this study, we employed CRISPR/Cas9 to generate stable, homozygous knockout lines of *OsHSBP1* (*Os09t0375100*) in the elite indica cultivar BT7. Our specific objectives were to: (1) evaluate whether complete knockout of *OsHSBP1* enhances heat tolerance at the seedling stage; (2) elucidate the physiological and molecular mechanisms underlying altered stress responses, including antioxidant enzyme activities and HSP gene expression; and (3) assess potential trade-offs between enhanced stress tolerance and key agronomic traits including yield components and grain quality under normal net-house conditions. This work provides definitive evidence for *OsHSBP1* function through complete gene knockout and delivers transgene-free, heat-tolerant germplasm with potential application for rice breeding programs.

## 2. Materials and Methods

### 2.1. Plant Material and Growth Condition

Wildtype (WT) rice cultivar BT7 (*Oryza sativa* L. ssp. *indica*) was provided by Thaibinh Seed Corporation (Thaibinh, Vietnam). The heat-tolerant cultivar RVT was provided by VinaSeed Corporation (Hanoi, Vietnam) and used as a positive control for stress tolerance assays. For seedling-stage experiments, germinated seeds were grown hydroponically in MS solution under controlled conditions of 16 h light/8 h dark photoperiod at 28 °C ± 2 °C with 70% relative humidity. For heat tolerance analysis at the seedling stage, 2-week-old plants were subjected to heat stress in a constant climate chamber at 45 °C for varying durations: 24 h for measurement of antioxidant enzyme activity and NBT staining, and 48 h for measurement of survival ratio. For gene expression analysis, 2-week-old plants were subjected to 42 °C condition for varying durations. For reproductive heat tolerance assessment and agronomic trait evaluation, germinated seeds were sown in soil pots and cultivated in a net-house. Plants at the heading stage were exposed to 38 °C for 2 days in a controlled climate chamber, and grain-filling rate was subsequently measured. Net-house cultivation conditions for both reproductive-stage experiments and transgenic/edited screening were as follows: 30 °C ± 2 °C for 14 h (light period) and 26 °C ± 2 °C for 10 h (dark period) with 80% relative humidity.

### 2.2. Gene Expression Analysis

Total RNA was extracted from 2-week-old rice seedlings using the GeneJET Plant RNA Purification Kit (Thermo Fisher Scientific, Waltham, MA, USA) according to the manufacturer’s instructions. One microgram of RNA was utilized with an oligo (dT) primer, followed by qPCR using gene-specific primers (Table S1). Quantitative real-time PCR was performed using gene-specific primers and SYBR Green PCR Master Mix (Thermo Fisher Scientific, Waltham, MA, USA) on a Bio-Rad CFX96 Real-Time PCR System (Bio-Rad Laboratories, Hercules, CA, USA) with 32 cycles for *OsHSBP1* and 29 cycles for *OsHSPs*. The *OsActin* gene was used as the internal reference [10]. Relative gene expression levels were calculated using the 2^−ΔΔCt^ method [17], where fold change values represent multiplicative factors relative to the control.

### 2.3. Generation of OsHSBP1 Mutant BT7 Rice Plants

**Figure 1:**
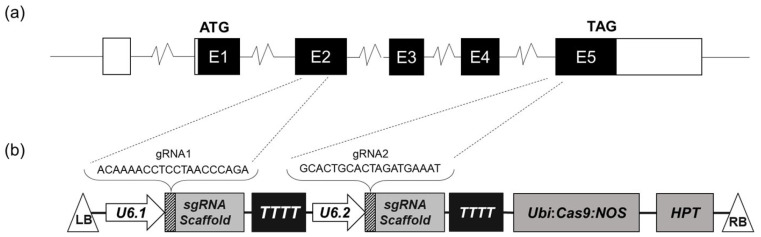
Construction of CRISPR/Cas9 vectors and transformation of BT7 rice. (**a**) Target sites of gRNA1 and gRNA2 in the *OsHSBP1* gene structure. Exons are shown as boxes, introns as lines. (**b**) Schematic diagram of the pCas9 binary vector containing two sgRNAs (gRNA1 and gRNA2) targeting the *OsHSBP1* gene. LB and RB: left and right borders of T-DNA; *Cas9*: codon-optimized *Cas9* gene; *Ubi*: maize *Ubiquitin* promoter; *NOS*: nopaline synthase terminator; *HPT*: hygromycin phosphotransferase gene for selection; *U6.1* and *U6.2*: promoters driving sgRNA expression.

Two single-guide RNAs (sgRNAs) were designed to target exon 5 of *OsHSBP1* (Figure 1a) using the online tools CRISPR-P v2.0 [18] and CCTop [19]. To minimize off-target effects, potential off-target sites were predicted and evaluated against the rice reference genome using CCTop with default parameters. The designed sgRNA sequences (Table S1) were commercially synthesized and cloned into the *Btg*ZI and *Bsa*I restriction sites of the entry vector pENTR4-sgRNA [20]. Both sgRNA expression cassettes were then transferred into the destination vector pCas9 [21] through Gateway LR Clonase-mediated recombination (Life Technologies, Carlsbad, CA, USA). The resulting binary vector (Figure 1b) was introduced into *Agrobacterium tumefaciens* strain EHA105 by electroporation for subsequent BT7 rice transformation as previously described [22]. Mature seeds of BT7 rice were dehusked, surface-sterilized, and cultured on callus induction medium (MS basal medium supplemented with 2.5 mg/L 2,4-dichlorophenoxyacetic acid) for 5–7 days. The induced calli were co-cultivated with *Agrobacterium* suspension on N6 medium containing 19.62 mg/L acetosyringone for 3 days in the dark. Following co-cultivation, the calli were transferred to selection medium (N6 basal medium supplemented with 200 mg/L cefotaxime, 200 mg/L vancomycin, and 50 mg/L hygromycin) and subcultured every 2 weeks. Hygromycin-resistant calli were transferred to shoot regeneration medium (MS basal medium containing 2 mg/L 6-benzylaminopurine, 0.2 mg/L 1-naphthaleneacetic acid, 0.5 mg/L kinetin, 20% (*v*/*v*) coconut water, and 20 mg/L hygromycin) under a 16 h photoperiod. Regenerated shoots were rooted on antibiotic-free MS medium, and the plantlets were acclimatized in a greenhouse.

The presence of transgene in T_0_ plants and its absence in T_1_ individuals were confirmed by PCR using *HPT*-, *Cas9*-, and sgRNA-specific primers (Table S1). A DNA fragment containing gRNA sites was amplified with specific primers Hs1-F/Hs1-R from all transgenic T_0_ or transgene-free T_1_ plants for *OsHSBP1* sequencing. *OsHSBP1* mutations were identified using the Degenerate Sequence Decoding method [23].

### 2.4. Physiological and Biochemical Parameters Measurements

For the survival ratio experiment, 2-week-old plants were subjected to 45 °C treatment for 48 h and then returned to normal conditions. The survival rate was observed after a 7-day recovery period, with plants showing regrowth and continued development considered as survivors.

For biochemical parameters measurement, 2-week-old plants were subjected to 45 °C treatment for 24 h. For enzyme assays, leaves were collected and ground in liquid nitrogen with phosphate buffer. Activities of catalase (CAT) and peroxidase (POD) were measured according to established methods [24]. CAT activity was determined by monitoring H_2_O_2_ decomposition at 240 nm, POD activity by guaiacol oxidation at 470 nm. Enzyme activities were expressed as U g^−1^ DW min^−1^. Malondialdehyde (MDA) content were determined spectrophotometrically as the methods described previously [25]. Lipid peroxidation was expressed as nmol MDA g^−1^ DW. For histochemical detection of superoxide radicals (O_2_^−^), leaves were immersed in NBT (nitro blue tetrazolium) solution (0.5 mg/mL in phosphate buffer, pH 7.5) and incubated at room temperature for 24 h in darkness. Leaves were then cleared in boiling ethanol (95%) to visualize the blue formazan precipitate.

### 2.5. Agronomic Parameter Assessment

Wildtype and mutant lines were grown in a net-house under controlled environmental conditions during the Autumn crop season of 2025 in Hanoi, Vietnam. The experiment was arranged in a randomized complete block design with three biological replicates.

Germination rate was evaluated by sowing 100 seeds per genotype in triplicate on moist filter paper at 28 °C. Germinated seeds (radicle emergence > 2 mm) were counted after 7 days, and germination percentage was calculated. For agronomic trait evaluation, five plants per genotype per replicate (15 plants total per genotype) were randomly selected. Measured traits included growth duration (day), plant height (cm), number of tillers per plant, number of panicles per plant, grain number per panicle, number of filled grains per panicle, thousand grain weight (g), individual grain yield per plant (g), and amylose content (%).

Amylose content was determined using the iodine colorimetric method [16]. Milled rice flour (100 mg) was gelatinized and reacted with iodine solution, and absorbance was measured at 620 nm.

### 2.6. Statistical Analysis

All experiments were performed with three biological replicates. Data are presented as mean ± standard deviation (SD). Statistical significance was determined by Student’s *t*-test for comparison between two groups or Duncan’s multiple range test for multiple comparisons using SPSS software v25.0. Differences were considered significant at *p* < 0.05.

## 3. Results

### 3.1. Heat Tolerance and OsHSBP1 Expression Profile of Wildtype

**Figure 2:**
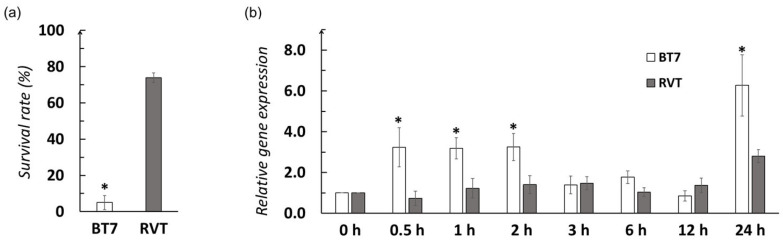
Heat stress tolerance and *OsHSBP1* expression pattern in wildtype BT7 rice. (**a**) Survival rate of BT7 and heat-tolerant cultivar RVT under heat stress (45 °C for 24 h followed by 7-day recovery). (**b**) Relative expression level of *OsHSBP1* in BT7 seedlings under heat stress (42 °C) at different time points (0, 0.5, 1, 2, 3, 6, 12, and 24 h). *OsActin* is used as an internal control. Data are presented as mean ± standard deviation (n = 3). Asterisks indicate significant differences compared to RVT (*p* < 0.05) by Student’s *t*-test.

The survival rate of BT7 seedlings after 45 °C heat stress for 24 h followed by 7-day recovery was 5.0%, significantly lower than the heat-tolerant cultivar RVT (73.8%) (Figure 2a). These results indicated that BT7 exhibited high sensitivity to heat stress compared to the stress-tolerant reference cultivar, demonstrating the need for genetic improvement of heat tolerance in this elite indica variety.

To investigate whether *OsHSBP1* is involved in heat stress response, RT-qPCR analysis was performed on wildtype BT7 seedlings under heat stress conditions. Under 42 °C heat treatment, *OsHSBP1* expression in BT7 showed an rapid induction during the first 2 h (3–3.5-fold increase, significantly higher than RVT), followed by a transient decline at 3–12 h to baseline levels similar to RVT, and then a dramatic surge at 24 h, reaching approximately 6-fold expression (Figure 2b). In contrast, the heat-tolerant cultivar RVT maintained relatively low and stable *OsHSBP1* expression levels throughout most time points, with only a moderate increase (~2.5-fold) at 24 h. Notably, BT7 exhibited significantly higher early-phase *OsHSBP1* induction (0.5–2 h) and late-phase expression (24 h) compared to RVT. This differential expression pattern between heat-sensitive and heat-tolerant cultivars suggests a potential role of *OsHSBP1* in modulating heat stress responses in rice, making it a promising candidate gene for functional characterization to improve heat tolerance.

### 3.2. Vector Construction and Development of BT7 Transgenic Plants

To functionally characterize *OsHSBP1*, a CRISPR/Cas9-mediated gene editing system was employed to generate knockout mutants in the BT7 background. Two sgRNAs (gRNA1 and gRNA2) were designed to target the Exon-5 coding sequence of BT7 *OsHSBP1* (Figure 1a). The sgRNAs were cloned into the binary vector pCas9 (Figure 1b), and the resulting constructs were introduced into BT7 embryogenic calluses via *Agrobacterium*-mediated transformation.

A total of 3600 mature embryos were used for callus induction, of which 3011 (83.64%) successfully formed embryogenic calluses (Table 1). Following co-cultivation with *Agrobacterium*, 2886 calluses (80.17%) survived the selection process. Among these, 989 calluses (27.47%) were successfully selected on hygromycin-containing medium, and 205 independent transgenic plants (5.69%) were regenerated from the selected calluses (Table 1). These T_0_ transgenic plants were transferred to the greenhouse for further analysis.

### 3.3. Genotyping of T_0_ Transgenic BT7 Plants

**Figure 3:**
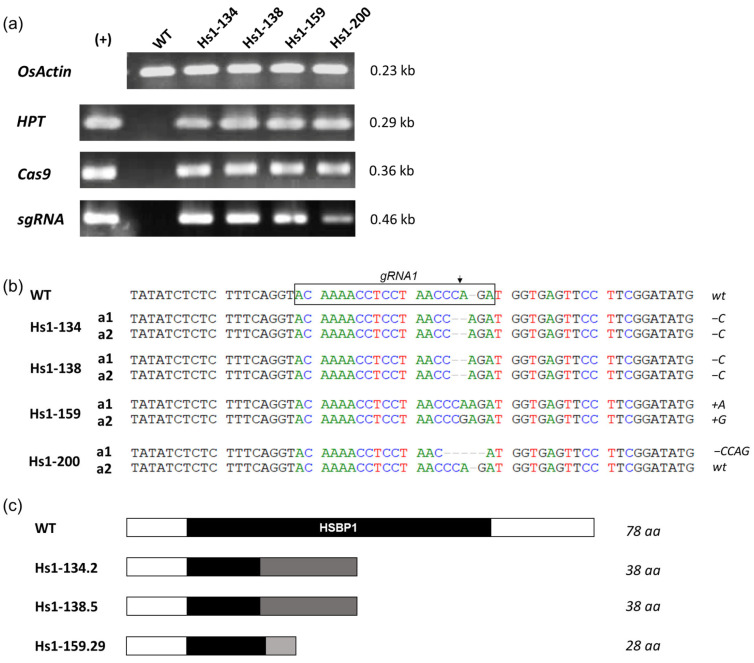
Molecular characterization of T_0_ transgenic plants and mutation identification at the *OsHSBP1* gRNA1 target site. (**a**) PCR screening of T_0_ transgenic lines using primers specific for *OsActin* (internal control), *HPT* (hygromycin resistance gene), *Cas9*, and sgRNA cassettes. (+): plasmid positive control; WT: wildtype BT7. (**b**) Sanger sequencing analysis of the gRNA1 target region in representative T_0_ mutant plants. The wildtype (WT) sequence is shown at the top with the gRNA1 target sequence in the box. The arrow indicates the predicted Cas9 cleavage site located 3 bp upstream of the PAM sequence. (a1, a2) two alleles of *OsHSBP1*. Deletions are indicated by dashes (-), and mutation types are noted on the right; (−): deletion; (+) insertion. (**c**) Schematic representation of predicted protein structures in selected T_1_ mutant lines derived from the corresponding T_0_ individuals. The functional HSBP1 domain is shown in black, and truncated or aberrant regions in gray. Polypeptide chain length (amino acids, aa) is indicated on the right.

PCR screening using three specific primer sets targeting *HPT*, *Cas9*, and sgRNA sequences identified 57/205 T_0_ plants (1.58% of initial embryos) carrying the complete T-DNA construct (Figure 3a). Among these PCR-positive plants, 27 independent individuals were randomly selected for sequencing analysis of the *OsHSBP1* target sites (Figure 3b).

Sequencing analysis revealed that 18 out of 27 plants (66.7%) carried mutations at the gRNA1 target site (Figure 3b), while no mutations were detected at the gRNA2 target site in any of the examined plants (Table 2). Among the 18 mutants, 10 plants (37.0%) were heterozygous, 2 (7.4%) were homozygous, and 6 (22.2%) were biallelic (Table 2). The remaining 9 plants (33.3%) showed no mutations and were classified as wildtype, indicating that the overall mutation efficiency at the gRNA1 target site was 66.7% among the transgene-positive plants.

Analysis of the mutation types showed that deletions were the most common, accounting for 19 out of 54 alleles (35.2%), followed by insertions (6/54, 11.1%) and substitutions (1/54, 1.9%) (Table 2). The modifications ranged from 1 to 21 base pairs (Figure S1). These mutations resulted in frameshift or in-frame alterations in the *OsHSBP1* coding sequence, potentially leading to loss of protein function.

These T_0_ mutants were grown in the greenhouse, and four high-seed-yielding individuals, including Hs1-134 (−C), Hs1-138 (−C), Hs1-159 (+A/+G), and Hs1-200 (−CCAG/*wt*) were selected for further analysis.

### 3.4. Development of Homozygous Transgene-Free OsHSBP1 Mutant Lines

To obtain stable homozygous mutant lines free of T-DNA insertion, the four selected T0 plants were allowed to self-pollinate for T_1_ generation analysis. A total of 238 T_1_ plants were initially screened by PCR using primers specific for *Cas9*, *HPT*, sgRNA, and the internal control *OsActin* to identify transgene-free individuals. The PCR screening identified 45 out of 238 plants (18.9%) that were negative for all T-DNA components (*Cas9*, *HPT*, and sgRNA) but positive for *OsActin*, indicating complete elimination of the transgene (Figure S2, Table S2).

These 45 transgene-free plants were subsequently subjected to Sanger sequencing of the *OsHSBP1* target region to identify homozygous mutants. Sequencing analysis revealed five independent homozygous knockout lines: Hs1-134.2 (−C), Hs1-134.60 (−C), Hs1-138.5 (−C), Hs1-138.8 (−C), and Hs1-159.29 (+G). Sequence alignment analysis showed that all five deletions resulted in frameshift mutations leading to premature stop codons and truncated OsHSBP1 proteins (Figure 3c and Figure S2). The predicted amino acid sequences confirmed that these mutations would produce non-functional proteins with significantly altered C-terminal regions or early termination, effectively creating null alleles of *OsHSBP1*.

Among the five homozygous mutant lines identified, three representative lines (Hs1-134.2, Hs1-138.5, and Hs1-159.29) derived from three independent T_0_ events were selected for seed propagation and phenotypic characterization in subsequent generations.

### 3.5. Mutation of OsHSBP1 Attenuated the Heat Stress Tolerance of BT7 Rice

**Figure 4:**
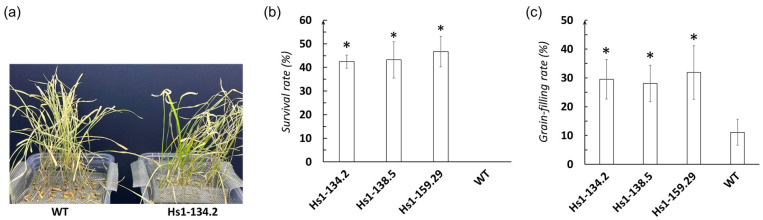
Effect of *OsHSBP1* knockout on heat stress tolerance in BT7 rice. (**a**) Representative images of WT and Hs1-134.2 mutant plants after heat stress treatment (45 °C) for 2 days. (**b**) Survival rates of homozygous mutant lines (Hs1-134.2, Hs1-138.5, and Hs1-159.29) and wild-type BT7 after heat stress (45 °C) followed by 7-day recovery. (**c**) Grain-filling rates of mutant and WT lines after 2-day heat treatment at 38 °C during the heading stage. Data are presented as mean ± standard deviation (n = 3 biological replicates with at least 30 plants per replicate). Asterisks indicate significant differences compared to WT (*p* < 0.05) by Student’s *t*-test.

To evaluate the effect of *OsHSBP1* knockout on abiotic stress tolerance, three homozygous mutant lines and wildtype BT7 were subjected to heat stress (45 °C) for 48 h followed by 7-day recovery periods. All three *OsHSBP1* knockout lines exhibited significantly increased tolerance compared to the wildtype (Figure 4a). In detail, the survival rates of Hs1-134.2, Hs1-138.5, and Hs1-159.29 were approximately 43–46%, all significantly higher (*p* < 0.05) than wildtype BT7 (0%) (Figure 4b). These results indicated that complete knockout of *OsHSBP1* resulted in dramatically improved tolerance to heat stress.

To further assess the impact of *OsHSBP1* knockout on reproductive heat tolerance, mutant lines and wildtype plants were exposed to 38 °C heat stress for 2 days during the heading stage. The grain-filling rates of the three mutant lines ranged from 28% to 32%, which were significantly higher (*p* < 0.05) than that of wildtype BT7 (approximately 11%) (Figure 4c). This substantial improvement in grain filling under heat stress during the critical reproductive stage demonstrated that *OsHSBP1* knockout not only enhanced vegetative heat tolerance but also protected reproductive development under heat stress conditions. Collectively, these findings suggest that OsHSBP1 plays a negative regulatory role in heat stress responses in BT7 rice, and its knockout represents a promising strategy for improving heat tolerance in elite rice cultivars.

### 3.6. Mutation of OsHSBP1 Attenuated Oxidative Stress Resistance in BT7 Rice Under Heat Stress

**Figure 5:**
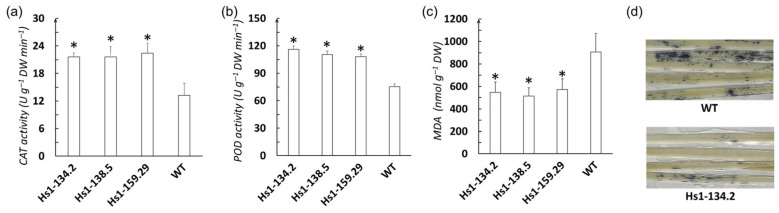
Antioxidant enzyme activities and oxidative stress markers in *OsHSBP1* knockout lines under heat stress. (**a**) Catalase (CAT) activity in three homozygous mutant lines (Hs1-134.2, Hs1-138.5, and Hs1-159.29) and wild-type BT7 after 24 h of heat stress at 45 °C. (**b**) Peroxidase (POD) activity under the same heat stress conditions. (**c**) Malondialdehyde (MDA) content. (**d**) Histochemical detection of superoxide radicals (O_2_^−^) by nitro blue tetrazolium (NBT) staining in leaves of WT and Hs1-134.2 after heat stress. Darker blue staining indicates higher O_2_^−^ accumulation. Data in panels (**a**–**c**) are presented as mean ± standard deviation (n = 3 biological replicates). Asterisks indicate significant differences compared to WT (* *p* < 0.05) by Student’s *t*-test.

To investigate the physiological mechanisms underlying the improved heat tolerance in *OsHSBP1* knockout lines, we measured antioxidant enzyme activities and oxidative damage markers under heat stress conditions (45 °C for 24 h). The catalase (CAT) activity in the three *OsHSBP1* knockout lines was significantly higher than wildtype, with mutant lines showing approximately 21–22 U g^−1^ DW min^−1^ compared to 14 U g^−1^ DW min^−1^ in wildtype (Figure 5a). Similarly, peroxidase (POD) activity in the mutant lines (105–115 U g^−1^ DW min^−1^) was significantly elevated compared to wildtype (70 U g^−1^ DW min^−1^) (Figure 5b). These results indicated that *OsHSBP1* knockout enhanced the antioxidant enzyme defense system under heat stress conditions.

Consistent with the elevated antioxidant enzyme activities, the malondialdehyde (MDA) content, a marker of lipid peroxidation, was significantly lower in the mutant lines (500–620 nmol g^−1^ DW) compared to wildtype (920 nmol g^−1^ DW) (Figure 5c), suggesting reduced membrane damage under heat stress. Nitro blue tetrazolium (NBT) staining further confirmed lower superoxide radical (O_2_^−^) accumulation in mutant leaves, displaying much lighter staining compared to the intense dark blue staining in wildtype leaves (Figure 5d). These findings collectively demonstrated that *OsHSBP1* knockout enhanced the plant’s ability to scavenge ROS through increased antioxidant enzyme activities, resulting in reduced oxidative damage and improved heat stress tolerance.

### 3.7. Expression of Heat-Specific HSP Genes in OsHSBP1 Mutant Rice

To further elucidate the molecular mechanism underlying the enhanced heat tolerance in *OsHSBP1* knockout lines, we examined the expression levels of key heat shock protein (HSP) genes under heat stress conditions. qRT-PCR analysis was performed on wildtype and three homozygous mutant lines (Hs1-134.2, Hs1-138.5, and Hs1-159.29) after 3 h of heat treatment at 42 °C.

**Figure 6:**
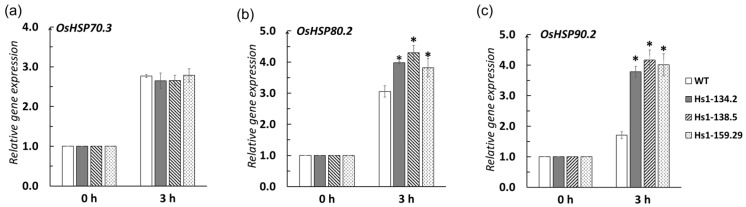
Expression pattern of *OsHSP* genes in *OsHSBP1* knockout BT7 lines under heat stress. Relative expression levels of (**a**) *OsHSP70.3*, (**b**) *OsHSP80.2*, and (**c**) *OsHSP90.2* in three homozygous mutant lines (Hs1-134.2, Hs1-138.5, and Hs1-159.29) and wild-type BT7 seedlings under normal conditions (0 h) and after 3 h of heat stress at 42 °C. Gene expression levels were normalized to the internal control *OsActin*, and the relative expression at 0 h was set to 1.0 for all genotypes. Data are presented as mean ± standard deviation (n = 3 biological replicates). Asterisks indicate significant differences compared to WT at 3 h time point (*p* < 0.05) by Student’s *t*-test.

After heat stress, differential expression patterns were observed among the examined *OsHSP* genes (Figure 6). *OsHSP70.3* showed no significant differences in expression levels between mutant lines and wildtype, with all lines exhibiting approximately 2.6–2.9-fold induction compared to the 0 h control (Figure 6a). In contrast, *OsHSP80.2* and *OsHSP90.2* displayed significantly enhanced expression in the *OsHSBP1* knockout lines. For *OsHSP80.2*, the mutant lines showed approximately 3.8–4.3-fold induction, which was significantly higher (*p* < 0.05) than wildtype (approximately 3.0 fold) (Figure 6b). Similarly, *OsHSP90.2* expression in the mutant lines reached approximately 3.8–4.2-fold induction, significantly exceeding the wildtype level of approximately 1.8-fold (Figure 6c).

These results demonstrated that loss of *OsHSBP1* function selectively enhanced the transcriptional activation of specific *HSP* genes, particularly *OsHSP80.2* and *OsHSP90.2*, under heat stress.

### 3.8. Agronomic Traits of OsHSBP1 Mutant Rice

**Figure 7:**
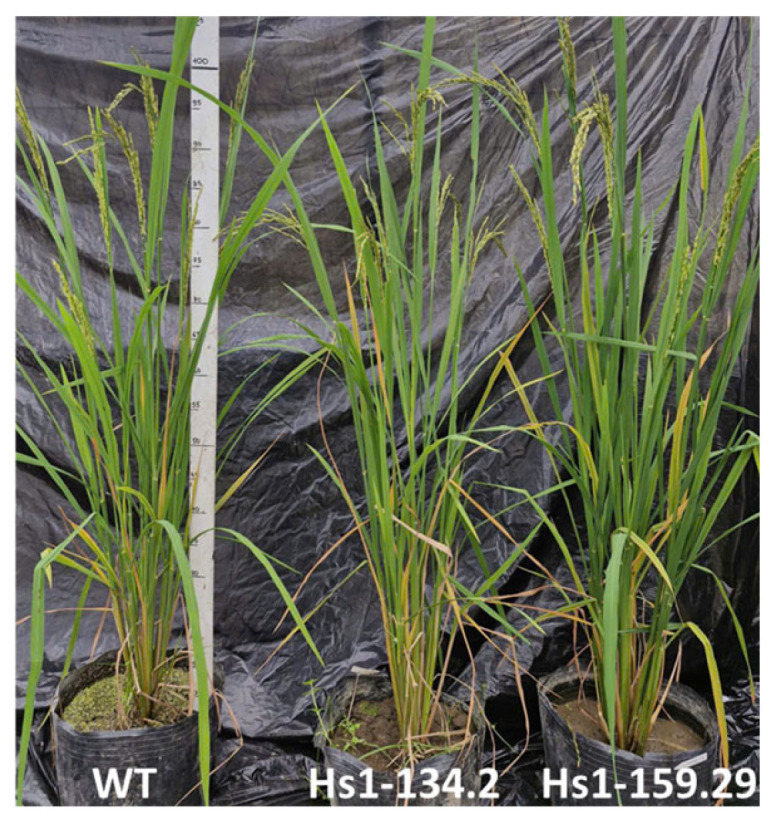
Plant morphology and panicle architecture of *OsHSBP1* knockout lines and wildtype at maturity under normal net-house conditions.

To evaluate whether *OsHSBP1* knockout affected plant growth and yield-related traits under normal green-house conditions, the three homozygous mutant lines and wildtype BT7 were grown in the net-house during the 2025 growing season in Hanoi, Vietnam (Figure 7).

There were no significant differences in germination rate among the three mutant lines (Hs1-134.2: 88.7%; Hs1-138.5: 90.0%; Hs1-159.29: 90.7%) and wildtype (90.0%) (Table 3). The growth duration of mutant lines ranged from 90.0 to 90.6 days, which was 4.6–5.2 days shorter than wildtype (95.2 days), although only the difference for Hs1-138.5 (90.0 days) and Hs1-159.29 (90.6 days) reached statistical significance (*p* < 0.05). Plant height and tiller number per plant showed no significant differences between mutant lines and wildtype, indicating that *OsHSBP1* knockout did not substantially affect vegetative growth under normal conditions.

For yield-related traits, the number of panicles per plant, grain number per panicle, and number of filled grains per panicle showed no significant differences between mutant lines and wildtype (Table 4). However, the number of filled grains per panicle in the mutant lines (117.6–125.3) showed a decreasing trend compared to wildtype (141.2), although the differences were not statistically significant. The 1000-grain weight remained comparable among all tested materials, ranging from 18.24 to 18.59 g. Consequently, the individual grain yield of mutant lines (8.65–10.25 g) showed no significant differences compared to wildtype (10.28 g). The amylose content, an important grain quality parameter, also remained unchanged in the mutant lines compared to wildtype (Table 4).

These results indicated that under normal growing conditions without stress, *OsHSBP1* knockout had minimal effects on major agronomic traits and yield performance in BT7 rice, suggesting that the primary function of *OsHSBP1* is related to stress responses rather than normal plant development.

## 4. Discussion

In this study, we successfully generated *OsHSBP1* knockout mutants in the elite indica rice cultivar BT7 using CRISPR/Cas9 technology and comprehensively evaluated their heat stress tolerance and agronomic performance. Our results revealed that *OsHSBP1* functions as a negative regulator of heat stress tolerance in rice, providing new insights for genetic improvement of climate-resilient rice varieties.

### 4.1. OsHSBP1 Acts as a Negative Regulator of Heat Stress Tolerance

The most striking finding of this study was that *OsHSBP1* knockout lines exhibited dramatically enhanced heat tolerance at the seedling stage. Under severe heat stress (45 °C for 48 h), the survival rates of mutant lines (43–46%) were substantially higher than wildtype (near 0%), clearly demonstrating that OsHSBP1 negatively regulates heat stress tolerance. This finding is consistent with its characterized role as a heat shock factor binding protein (HSBP) that attenuates heat shock response (HSR) activation.

At the molecular level, HSBPs function as negative regulators of HSR by binding to active HSF trimers and preventing their DNA-binding and transcriptional activities [4,5]. The interaction between HSBP and HSF trimers promotes dissociation of the trimeric complex, thereby terminating the transcriptional activation of heat shock protein genes [10]. By removing this negative regulator through CRISPR/Cas9-mediated knockout, the mutant plants likely maintained higher and more sustained HSF activity under heat stress, leading to enhanced expression of downstream stress-responsive genes and improved cellular protection.

The expression pattern of *OsHSBP1* under heat stress provides additional insights into its regulatory function. We observed a biphasic response (Figure 2b) which suggests that *OsHSBP1* plays distinct roles at different phases of heat stress response. The early induction (0–2 h) may function to prevent excessive HSR activation and energy depletion during the initial heat shock [26]. The mid-phase suppression (3–12 h) may result from several interconnected mechanisms: (i) autoregulatory negative feedback, where accumulated OsHSBP1 protein inhibits HSF activity, which in turn reduces *OsHSBP1* transcription; (ii) chromatin remodeling and epigenetic regulation that transiently suppress *OsHSBP1* transcription during the adaptive phase; and (iii) competition for limited HSF transcription factors among multiple heat-responsive genes, where *OsHSBP1* expression may be temporarily deprioritized in favor of protective genes such as HSPs and antioxidant enzymes. The strong late-phase expression (24 h) could be involved in attenuating HSR during the recovery phase to restore normal cellular homeostasis [27]. This dynamic regulation ensures a balanced stress response that protects cells without constitutively activating energy-consuming defense pathways. Elucidating the specific upstream regulators—including transcription factors, chromatin modifiers, and signaling components—that govern this temporal expression pattern represents an important direction for future research and would provide deeper mechanistic insights into HSR fine-tuning in rice.

### 4.2. Enhanced HSP Expression and Antioxidant Capacity

Our molecular analysis revealed that the enhanced heat tolerance in *OsHSBP1* knockout lines is underpinned by two interconnected mechanisms: elevated expression of specific heat shock protein genes and enhanced antioxidant defense capacity.

Several *HSP* genes have been shown to exhibit strong upregulation under heat stress conditions in rice, including *OsHSP70.3* [28], *OsHSP80.2* [29] and *OsHSP90.2* [30]. Our gene expression analysis demonstrated that *OsHSBP1* knockout selectively enhanced the expression of these *HSP* genes under heat stress (Figure 7). This selective enhancement is particularly noteworthy, as it suggests that *OsHSBP1* does not uniformly repress all HSR genes, but rather preferentially regulates specific HSP subsets. HSP90 family proteins function as molecular chaperones critical for protein folding, stability, and signal transduction under stress conditions [31], while HSP80 proteins play important roles in stress response and protein quality control [29]. The preferential upregulation of these specific HSPs likely contributed to improved protein homeostasis and enhanced cellular protection in the knockout lines. Notably, this finding differs from the report by Rana et al. (2012) [10], who observed significant upregulation of *OsHSP70* in their *OsHSBP1* knockdown lines. However, this apparent discrepancy may be explained by the complexity of the *HSP70* gene family in rice. The *OsHSP70* family comprises at least 32 members with highly diverse expression patterns under heat stress conditions [28], and Rana et al. did not specify which *OsHSP70* member were analyzed in their study. In our study, we specifically examined *OsHSP70.3*, which showed no differential expression between mutant and wildtype lines. It is plausible that other *OsHSP70* family members may exhibit different responses to *OsHSBP1* disruption. Similarly, while Rana et al. reported upregulation of small *HSPs* (*OsHSP16.9* and *OsHSP17*.5) in their knockdown lines, we focused our analysis on *HSP80* and *HSP90* family members, which are known to play critical roles as molecular chaperones in protein quality control and stress signaling. A comprehensive expression profiling of the entire *HSP* gene family in *OsHSBP1* knockout lines would provide a more complete picture of the regulatory network, and represents an important direction for future research. This selective enhancement is particularly noteworthy, as it suggests that OsHSBP1 does not uniformly repress all *HSP* genes, but rather preferentially regulates specific *HSP* subsets. The preferential upregulation of *OsHSP90.2* and *OsHSP80.2* in the knockout lines likely contributed to improved protein homeostasis and enhanced cellular protection under heat stress [29,30].

At the physiological level, the mutant lines showed significantly enhanced antioxidant enzyme activities, including CAT and POD, under heat stress (Figure 5a,b). This enhanced enzymatic activity was accompanied by substantially reduced oxidative damage, as evidenced by lower MDA content and reduced superoxide radical accumulation visualized by NBT staining (Figure 5c,d). The mechanistic link between *OsHSBP1* knockout and elevated antioxidant capacity can be explained through several interconnected pathways. First, upregulated HSPs, particularly HSP90 and HSP80, act as molecular chaperones that protect antioxidant enzymes from heat-induced denaturation and aggregation, thereby maintaining their catalytic activity under stress conditions [31]. Second, HSPs may facilitate the proper folding and assembly of newly synthesized antioxidant enzymes, ensuring continuous replenishment of the ROS scavenging machinery. Third, emerging evidence suggests that HSPs can modulate the activity of transcription factors that regulate antioxidant gene expression, creating a positive feedback loop between protein homeostasis and oxidative stress defense [32]. The coordinated upregulation of antioxidant enzymes likely resulted from enhanced expression of *HSPs* and other stress-responsive genes in the absence of *OsHSBP1*-mediated repression. Heat stress typically induces excessive accumulation of reactive oxygen species (ROS), leading to oxidative damage to cellular membranes, proteins, and nucleic acids [32]. Under heat stress, ROS production overwhelms the basal antioxidant capacity, necessitating rapid upregulation of ROS-scavenging enzymes. In *OsHSBP1* knockout lines, the derepression of HSF activity enables coordinated activation of both *HSP* genes and antioxidant defense genes, creating a synergistic protective effect. The ability to rapidly activate antioxidant defense systems is therefore crucial for plant survival under heat stress [33]. Our results demonstrate that eliminating *OsHSBP1* enables more robust and sustained activation of these protective mechanisms, thereby minimizing oxidative damage and maintaining cellular integrity during heat stress.

### 4.3. Minimal Trade-Offs Between Heat Tolerance and Agronomic Performance

A critical concern in developing stress-tolerant crop varieties is the potential trade-off between enhanced stress tolerance and yield performance under normal growing conditions. Such trade-offs often arise because constitutive activation of stress defense pathways can divert resources away from growth and reproduction [34]. Our comprehensive agronomic evaluation revealed that *OsHSBP1* knockout had minimal negative effects on major agronomic traits under normal greenhouse conditions.

Although the mutant lines showed slightly shorter growth duration compared to wildtype (Table 3), other key agronomic traits remained largely unaffected. Plant height, tiller number, panicle number, grain number per panicle, thousand grain weight, individual grain yield, and grain quality (amylose content) showed no significant differences between mutant lines and wildtype (Table 3 and Table 4). The mutant lines exhibited a non-significant trend toward reduced filled grain number per panicle, but this did not translate into significant yield reduction, as the overall grain yield per plant remained comparable to wildtype.

Our findings partially align with, but also differ from, the previous report by Rana et al. (2012) [10], who used RNA interference (RNAi) to knock down *OsHSBP1* expression in rice. Similarly to their findings, we observed a significant reduction in growth duration and no change in plant height. However, while Rana et al. reported a significant decrease in filled grain percentage in their RNAi lines, our CRISPR/Cas9 knockout mutants showed only a non-significant trend toward reduced filled grain number. This difference may be attributed to several factors. First, the genetic approaches differ fundamentally: RNAi typically achieves partial gene suppression with variable knockdown efficiency across different tissues and developmental stages, whereas CRISPR/Cas9-mediated knockout results in complete and stable loss of gene function. The partial suppression in RNAi lines might trigger compensatory mechanisms or pleiotropic effects that differ from complete knockout. Second, genetic background differences between the rice cultivars used in the two studies (Zhonghua11 in Rana’s study versus BT7 in our study) could contribute to differential responses to *OsHSBP1* disruption. Additionally, differences in experimental conditions between the two studies may also contribute to the observed phenotypic variations.

This finding is particularly encouraging for practical breeding applications, as it suggests that *OsHSBP1* knockout can enhance seedling heat tolerance without substantially compromising yield potential under favorable growing conditions. The minimal impact on agronomic traits indicates that OsHSBP1’s primary function is related to stress response regulation rather than essential developmental processes. This makes *OsHSBP1* an attractive target for genetic improvement of heat tolerance in rice.

### 4.4. Future Perspectives and Breeding Applications

Our results demonstrate that CRISPR/Cas9-mediated knockout of *OsHSBP1* is a promising strategy for improving heat tolerance in elite rice cultivars. The transgene-free homozygous lines generated in this study represent valuable germplasm resources that can be directly integrated into breeding programs without the regulatory hurdles associated with transgenic crops. These knockout alleles are particularly valuable for developing rice varieties adapted to regions experiencing increasingly frequent heat waves due to climate change.

Several research directions merit further investigation. First, the molecular mechanisms by which *OsHSBP1* selectively regulates specific *HSP* genes need to be elucidated. Understanding the HSF-HSBP interaction dynamics and identifying the specific HSF isoforms regulated by *OsHSBP1* would provide insights for fine-tuning stress responses. Second, comprehensive field evaluation under natural heat stress conditions across multiple locations and growing seasons is essential to validate the breeding value of *OsHSBP1* knockout alleles and assess their performance under diverse environmental conditions. Third, generating and characterizing *OsHSBP1/OsHSBP2* double knockout mutants would clarify the extent of functional redundancy between these two genes and determine whether simultaneous knockout provides additive or synergistic effects on heat tolerance. Fourth, the applicability of *OsHSBP1* knockout should be tested across different rice genetic backgrounds, including both indica and japonica subspecies, to assess the generality of its effects. This consideration is particularly important given that indica and japonica rice subspecies exhibit fundamental differences in heat stress tolerance and employ distinct molecular response pathways. Indica rice, adapted to tropical and subtropical environments, generally shows superior thermotolerance compared to temperate-adapted japonica rice. Recent comparative studies have revealed subspecies-specific molecular mechanisms underlying heat tolerance, such as SLG1, which is naturally differentiated between indica and japonica and contributes to higher thermotolerance in indica through enhanced tRNA 2-thiolation [35]. These findings suggest that heat stress response mechanisms may not be universally conserved across subspecies. Therefore, validating OsHSBP1 function in representative japonica cultivars would be valuable to determine whether this negative regulatory mechanism operates similarly in both subspecies or represents an indica-specific adaptation. Such comparative studies would not only enhance our fundamental understanding of heat stress biology but also inform targeted breeding strategies for both subtropical (indica-growing) and temperate (japonica-growing) regions facing increasing heat stress due to climate change. Future work should investigate potential allelic variations in *OsHSBP1* between subspecies and assess whether knockout confers similar benefits in japonica cultivars.

Finally, combining *OsHSBP1* knockout with other stress tolerance genes through gene pyramiding or molecular breeding approaches may further enhance the magnitude and stability of heat tolerance. For example, stacking *OsHSBP1* knockout with positive regulators of stress tolerance such as *OsDREB* genes or genes encoding osmotic adjustment compounds could provide more robust and multifaceted stress tolerance [36]. Additionally, leveraging genomic selection and high-throughput phenotyping technologies could accelerate the introgression of *OsHSBP1* knockout alleles into diverse elite cultivars adapted to different growing regions [37].

## 5. Conclusions

This study demonstrated that *OsHSBP1* functions as a negative regulator of heat stress tolerance in rice. CRISPR/Cas9-mediated knockout of *OsHSBP1* in elite indica cultivar BT7 dramatically improved seedling survival under severe stress conditions (43–46% vs. near 0% in wildtype under heat stress) through enhanced antioxidant enzyme activities and reduced oxidative damage. Importantly, the improved stress tolerance was achieved without significant yield penalty under normal conditions, indicating that *OsHSBP1* primarily functions in stress response rather than normal development.

The transgene-free homozygous knockout lines generated in this study represent valuable germplasm for breeding climate-resilient rice varieties. Our findings demonstrate that targeting negative regulators of stress pathways through precision gene editing is a promising strategy for improving crop resilience to climate change while maintaining yield potential.

## Figures and Tables

**Table 1:** Efficiency of *Agrobacterium*-mediated transformation in BT7 rice.

Experiment	No. of Sample	Rate (%) ^1^
Mature embryo	3600	100.00
Callus	3011	83.64
Co-cultivation	2886	80.17
Selection	989	27.47
Regeneration	205	5.69
PCR ^2^	57	1.58

^1^ Rate (%) was calculated relative to the initial number of embryos. ^2^ PCR screening was performed using three specific primer sets targeting *Cas9*, *HPT*, and sgRNA sequences to confirm the presence of complete T-DNA constructs in regenerated plant.

**Table 2:** Mutation types and zygosity of *OsHSBP1* at the target sites in T_0_ transgenic plants.

Position	*OsHSBP1* Genotype ^1^				Mutation Type ^2^		
	Heterozygous	Homozygous	Bialellic	*wt*	Deletion	Insertion	Substitution
gRNA1	10/27	2/27	6/27	9/27	19/54	6/54	1/54
gRNA2	0/27	0/27	0/27	27/27	0/54	0/54	0/54
gRNA1 –2	10/27	2/27	6/27	9/27	19/54	6/54	1/54

^1^ Number of genotypes/total number of genotypes. ^2^ Number of allele mutation type/total number of all alleles.

**Table 3:** Vegetative growth characteristics of *OsHSBP1* knockout lines under normal net-house conditions.

Lines	Germination Rate (%)	Growth Duration (Day)	Plant Height (cm)	No. of Tiller per Plant
Hs1-134.2	88.67 ± 3.06 ^a^	90.0 ± 1.87 ^a^	108.0 ± 8.77 ^a^	6.6 ± 1.14 ^a^
Hs1-138.5	90.00 ± 3.06 ^a^	90.0 ± 3.32 ^ab^	117.4 ± 7.96 ^a^	7.0 ± 1.00 ^a^
Hs1-159.29	90.67 ± 3.06 ^a^	90.6 ± 2.70 ^a^	116.6 ± 4.10 ^a^	6.4 ± 1.14 ^a^
WT	90.00 ± 3.06 ^a^	95.2 ± 2.77 ^b^	119.4 ± 3.36 ^a^	6.8 ± 1.30 ^a^

Data are presented as mean ± standard deviation (n = 3 biological replicates with 5 plants per replicate). Different letters indicate significant differences at *p* < 0.05 by Duncan’s multiple range test. Plants were grown in the net-house during the 2025 growing season in Hanoi, Vietnam.

**Table 4:** Yield components and grain quality traits of *OsHSBP1* knockout lines under normal net-house conditions.

Lines	No. of Panicles	No. of Grains per Panicle	No. of Filled Grains per Panicle	P1000 (g)	Individual Yield (gr)	Amylose Content (%)
Hs1-134.2	3.8 ± 0.84 ^a^	165.3 ± 17.09 ^a^	125.3 ± 11.32 ^a^	18.45 ± 0.12 ^a^	8.65 ± 1.10 ^a^	17.92 ± 0.64 ^a^
Hs1-138.5	4.6 ± 0.55 ^a^	156.3 ± 11.22 ^a^	120.6 ± 9.54 ^a^	18.59 ± 0.18 ^a^	10.25 ± 0.79 ^a^	17.61 ± 0.51 ^a^
Hs1-159.29	4.0 ± 0.71 ^a^	156.8 ± 6.34 ^a^	117.6 ± 9.91 ^a^	18.58 ± 0.33 ^a^	8.77 ± 1.85 ^a^	18.15 ± 0.14 ^a^
WT	3.4 ± 0.55 ^a^	155.0 ± 8.36 ^a^	141.24 ± 8.57 ^a^	18.24 ± 0.21 ^a^	10.28 ± 0.96 ^a^	18.15 ± 0.88 ^a^

Data are presented as mean ± standard deviation (n = 3 biological replicates with 5 plants per replicate). Different letters indicate significant differences at *p* < 0.05 by Duncan’s multiple range test. P1000: 1000-grain weigh. Plants were grown in the net-house during the 2025 growing season in Hanoi, Vietnam.

## Data Availability

The original contributions presented in this study are included in the article/Supplementary Material. Further inquiries can be directed to the corresponding author(s).

## Supplementary Materials

The following supporting information can be downloaded at: https://www.mdpi.com/article/10.3390/biotech15010013/s1. Table S1: The primers used in study; Table S2: Segregation analysis of T-DNA components and identification of homozygous mutants in the T_1_ generation; Figure S1: Sanger sequencing analysis of the gRNA1 target region in representative T_0_ mutant plants; Figure S2. Identification of transgene-free homozygous *OsHSBP1* mutant lines in the T_1_ generation.
