# Supplementary material for: CRISPR/Cas9-Mediated Knockout of OsHSBP1 Confers Heat Tolerance to Bacthom 7 Elite Rice Cultivar
Source: BioTech (Basel). 2026 Feb 4;15(1):13. doi: 10.3390/biotech15010013 (PMC12922113; doi:10.3390/biotech15010013)
Supplement: Supplementary file 1 [file biotech-15-00013-s001.zip › biotech-4079785-supplementary.pdf]

Table S1. The primers used in study.

Prime name	Sequence (5'-3')	Gene/vector	Ref.
Actin-F	TGATGGTGTCAGCCACACT	*OsActin*	[16]
Actin-R	TGGTCTTGGCAGTCTCCATT		
HSBP1-F	TGTTTTGCTTTGGAGGTAGCA	*OsHSBP1*	
HSBP1-R	TGTAATGCAACGACAACCCT		
HPT-F	AAACTGTGATGGACGACACCGT	*HPT*	[16]
HPT-R	GTGGCGATCCTGCAAGCTCC		
Ubi-F	CCCTGCCTTCATACGCTATT	pCas9	[16]
Cas9-t-R	GCCTCGGCTGTCTCGCCA		
BtgZI-gRNA1-F	TGTT[GACAAAACCTCCTAACCCAGA]	gRNA1	
BtgZI-gRNA1-R	AAAC[TCTGGGTTAGGAGGTTTTGTC]		
BsaI-gRNA2-F	GTGT[GCACTGCACTAGATGAAAT]	gRNA2	
BsaI-gRNA2-R	AAAC[ATTTCATCTAGTGCAGTGC]		
RT-HSBP1-F	TCAATCCTCACCCGGGAAATCG	*OsHSBP1*	[10]
RT-HSBP1-R	CACAATGTTCTGGGACATAG		
RT-HSP70.3-F	TGAGGGAGGCTGAGCTACAT	*OsHSP70.3*	[28]
RT-HSP70.3-R	CAACTTCCTATTACATGAAC		
RT-HSP80.2-F	CGACGACGAGCAGTATGT	*OsHSP80.2*	[26]
RT-HSP80.2-R	CCAGATGTTCCTCCCAGT		
RT-HSP90.2-F	TCATCACCTCCACTCTCTGC	*OsHSP90.2*	[25]
RT-HSP90.2-R	ATCAGCTTTGGACTCCCAGA		

Table S2. Segregation analysis of T-DNA components and identification of homozygous mutants in the T_1 generation.

T_0 Plant	Total T_1 plants screened	T-DNA free	Homozygous mutant
Hs1-134	60	15	2
Hs1-138	62	15	2
Hs1-159	58	14	1
Hs1-200	58	1	0
Total	238	45	5

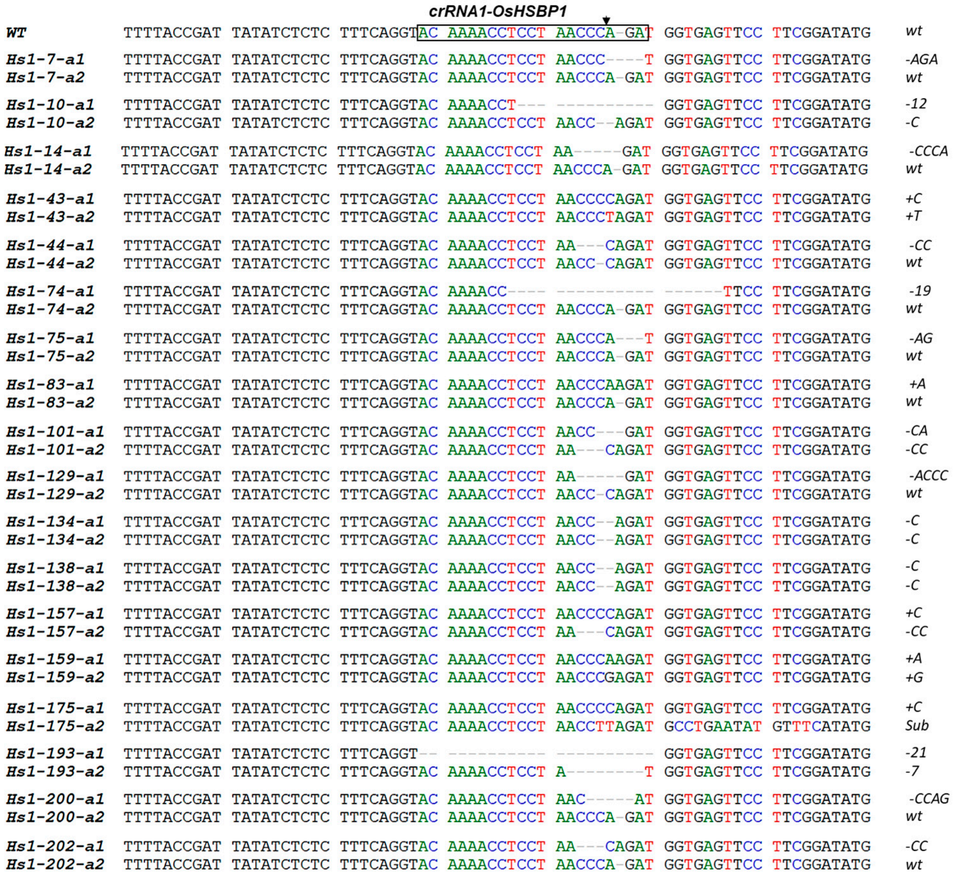

Figure S1. Sanger sequencing analysis of the gRNA1 target region in representative T_0 mutant plants.

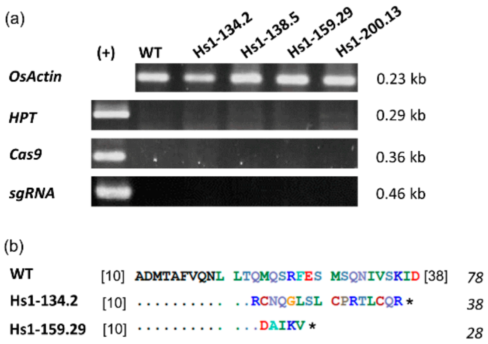

Figure S2. Identification of transgene-free homozygous *OsHSBP1* mutant lines in the T_1 generation. (a) PCR screening of T_1 plants for T-DNA elimination using primers specific for *Cas9*, *HPT*, sgRNA, and internal control *OsActin*. (+): plasmid positive control; WT: wild-type BT7. (b) Predicted amino acid sequence alignment of wild-type *OsHSBP1* and homozygous mutant lines. Deletions at the gRNA1 target site resulted in frameshift mutations and premature stop codons (marked with asterisks), leading to truncated proteins. The mutation size (in amino acid residue) is indicated on the right.
